# Pheochromocytoma in an Ectopic Adrenal Gland

**DOI:** 10.7759/cureus.40068

**Published:** 2023-06-06

**Authors:** Salma Wahbi, Siham B Cherkaoui, Hayat Aynaou, Houda Salhi, Hanan El Ouahabi

**Affiliations:** 1 Department of Endocrinology, Diabetology, Metabolic Diseases and Nutrition, Hassan II University Hospital, Fez, MAR; 2 Department of Endocrinology, Diabetology, Metabolic Diseases and Nutrition, Hassan II University Hospital, Fes, MAR

**Keywords:** retro-hepatic mass, ectopic adrenal gland, ectopic adrenal tissue, pheochromocytoma, paraganglioma

## Abstract

A pheochromocytoma is an uncommon tumor that originates from the chromaffin cells of the adrenal medulla. Also, adrenal tissue not located in its typical position is referred to as ectopic adrenal tissue. It is relatively uncommon in adults and is usually asymptomatic. Therefore, a pheochromocytoma arising from ectopic adrenal tissue is even a rarer finding and presents as a unique diagnostic challenge.

A 20-year-old man presented with vague abdominal pain, and upon imaging, a mass located behind the liver was initially discovered. Subsequently, it was identified as a mass growing in an ectopic adrenal gland. He underwent exploratory laparotomy and resection of the mass. A pheochromocytoma in an ectopic adrenal gland was confirmed by histopathology.

## Introduction

In 1740, Morgagni was the first to report ectopic adrenal tissue as a yellowish nodule located in the spermatic cord of a child [1]. Since then, several cases have documented the presence of ectopic adrenal tissue in various locations [2-6].

Typically, ectopic adrenal tissue is located near the adrenal glands and is found along the path of their descent due to the close association between the adrenal and urogenital primordial. However, there have also been reports of ectopic adrenal tissue in the gallbladder and liver [7,8].

To the best of our knowledge, there is only one reported case of pheochromocytoma in an ectopic adrenal gland in the literature, which occurred in Japan [9].

## Case presentation

We report a case of a 20-year-old male with an insignificant medical history, who was presented to the general surgery department with a one-year history of abdominal pain, weight loss, and diminished appetite but no other notable digestive symptoms.

**Figure 1:**
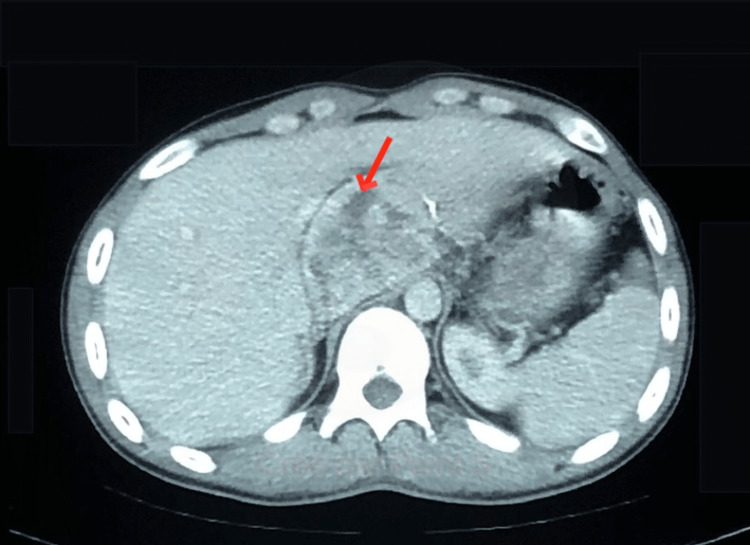
Cross section of an MRI revealing a 9.7-cm-wide retro-hepatic mass abutting the right adrenal gland MRI, magnetic resonance imaging.

Initial enhanced magnetic resonance imaging (MRI), which was requested by the surgery department due to persistent abdominal pain, revealed a 76×46×97 mm retro-hepatic mass abutting the right adrenal gland, compatible with a paraganglioma (Figure 1).

A hormonal workup, including 24-hour urinary methoxylated derivatives, was performed and showed a two-times increase in urinary normetanephrine with normal urinary metanephrine and dopamine levels.

**Figure 2:**
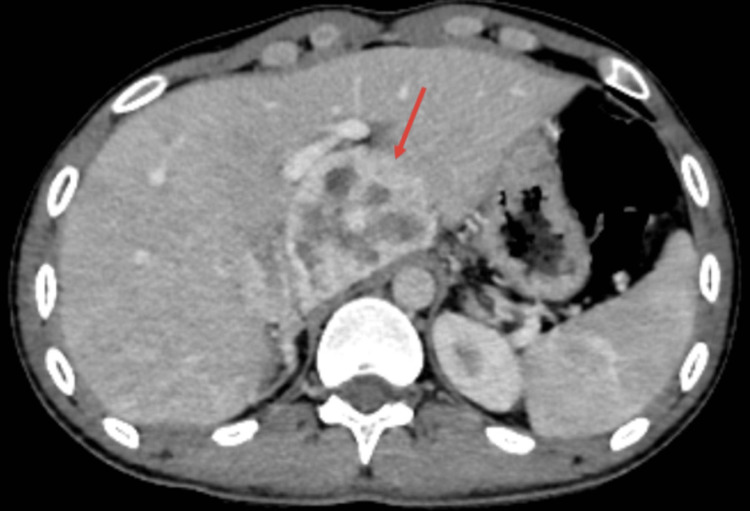
Cross section of CT scan imaging showing a tissular mass of the right adrenal gland CT, computed tomography.

A computed tomography (CT) body scan revealed a 75×43 mm encapsulated tissue mass in close intimacy to the right adrenal gland (Figure 2), suggesting a paraganglioma, with no other localization.

**Figure 3:**
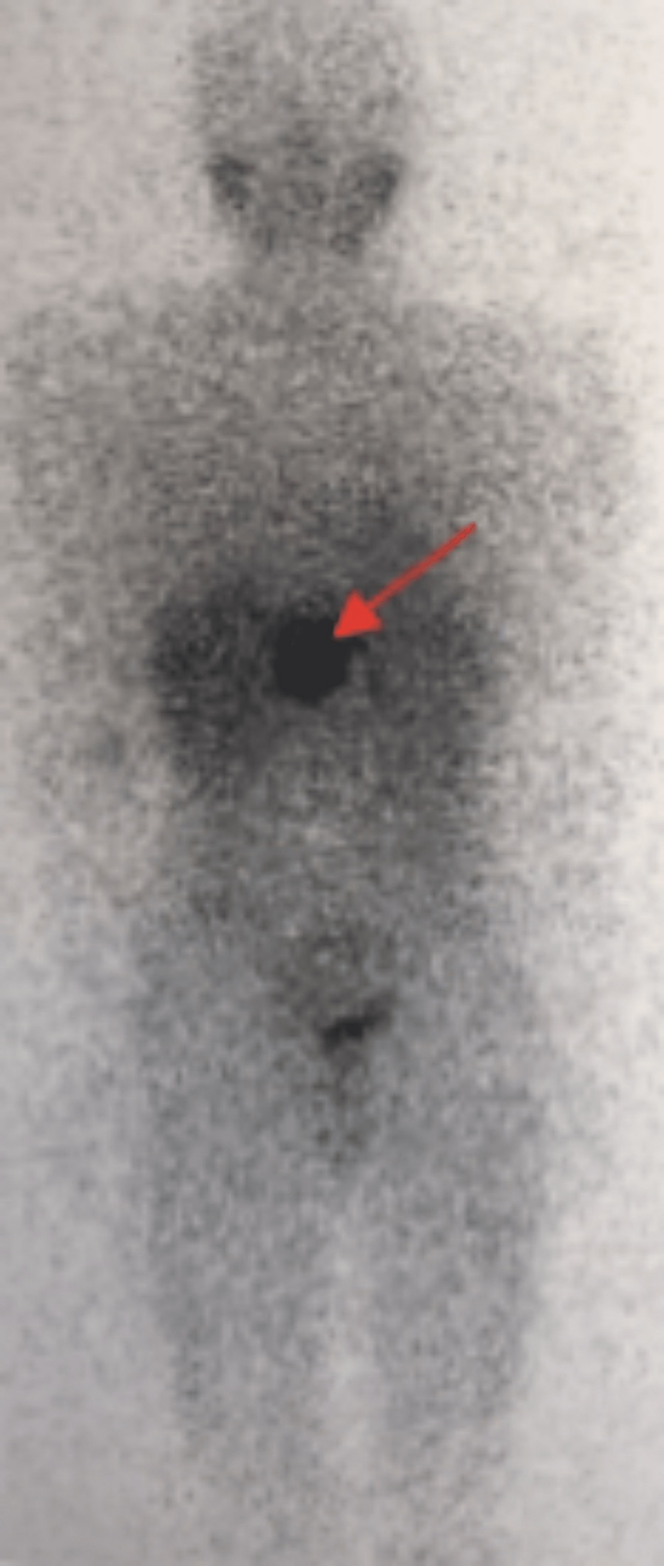
MIBG scintigraphy shows clear fixation corresponding to the retro-hepatic mass described in the MRI MIBG, metaiodobenzylguanidine; MRI, magnetic resonance imaging.

Metaiodobenzylguanidine (MIBG) scintigraphy demonstrated a clear fixation in the epigastric region corresponding to a hepatic retro-hilar paraganglioma (Figure 3).

Molecular analysis of the von Hippel-Lindau (VHL) gene and the RET gene did not detect any anomaly. A succinate dehydrogenase complex iron sulfur subunit B (SDHB) study was requested.

The patient was then taken to the operating room. There was no indication of damage to the right kidney or its blood vessels, and the left adrenal gland remained intact. The right adrenal gland for its part was not seen. The tumor was meticulously removed in one piece, which appeared to be a right adrenal tumor located in an ectopic position.

The postoperative period was uneventful, and the patient was discharged on the eighth day.

The test result for postoperative urinary catecholamines was negative.

**Figure 4:**
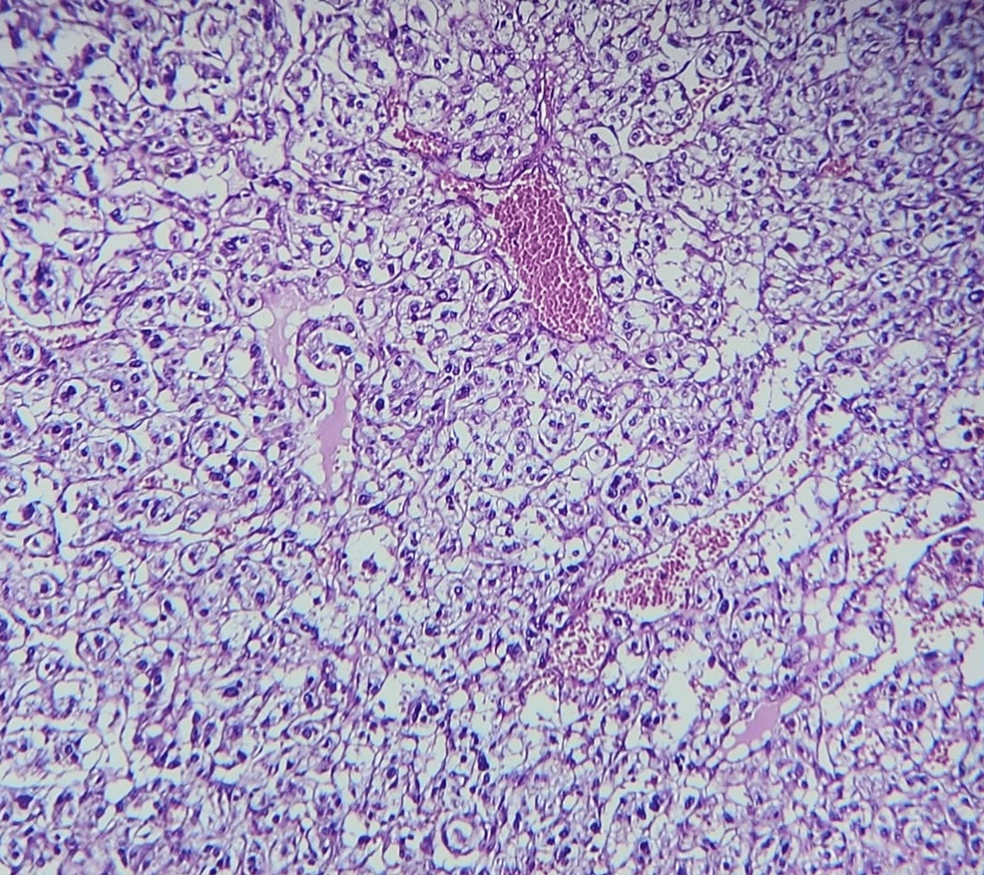
Hematoxylin-eosin stain revealing a nested architecture called “zellballen”

**Figure 5:**
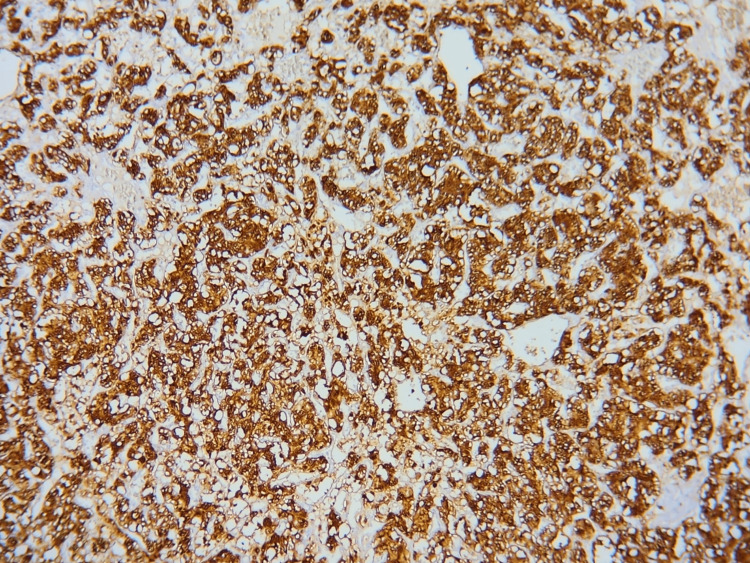
Immunohistochemistry showing clear positivity for chromogranin A

**Figure 6:**
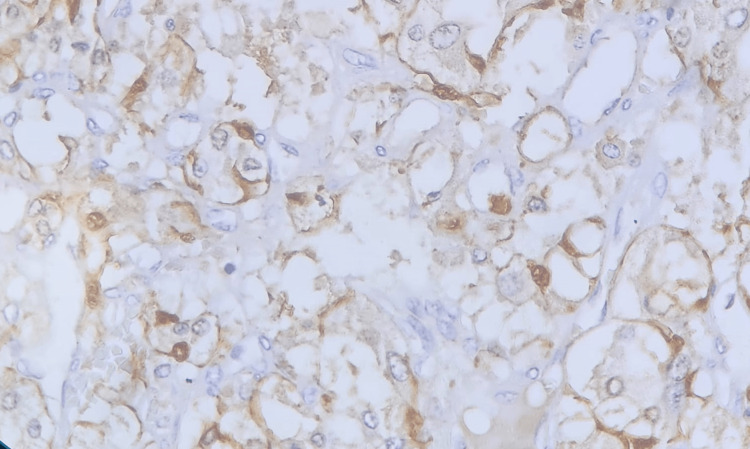
Sustentacular cells stained with S100 protein immunostain

Grossly, the mass measured 8×7×4 cm and was blackish in appearance, homogeneous, and surrounded by a capsule. Histopathology and immunohistochemistry initially suggested a paraganglioma but then concluded to a pheochromocytoma arising from an ectopic adrenal gland (Figures 4, 5, 6). 

The diagnosis of a pheochromocytoma was supported by the presence of a small amount of normal adrenal tissue observed in pathological examination. As the left adrenal gland was not affected, it is thought that the pheochromocytoma originated from a right adrenal gland located in an ectopic position.

The patient has been under surveillance and has not shown evidence of recurrence.

## Discussion

Ectopic adrenal tissue is not as uncommon as thought to be; it affects around 6% of the population. Morgagni initially observed this condition in 1740, describing adrenal tissue in a child's spermatic cord as little yellow nodules with an adipose-like appearance [1].

Abnormal adrenal gland organogenesis can cause ectopic adrenal tissue [10]. To better understand adrenal ectopy, we must first acquire more knowledge on the embryology of the adrenal glands. 

The adrenal cortex develops from the mesoderm between the fourth and fifth weeks of pregnancy. Ectopic cortical tissue may be present anywhere along the path of gonadal descent due to the physical closeness of the developing gonads and the cortex. The medulla develops from the ectoderm of the neural crest and infiltrates the developing cortex, eventually transforming into the definitive medulla during embryonic growth.

The majority of cases of ectopic adrenal tissue are discovered by chance. Moreover, most cases of ectopic adrenal tissue in the literature were observed in infants [11].

In our case, the tissue was obtained from a 20-year-old adult, which is unusual given that ectopic adrenal tissue usually regresses in early infancy. Falls [2] discovered 13 nodules in the wide ligaments of 11 women over the course of 14 months, implying that this anomaly may be more prevalent than previously thought. Nevertheless, a retro-hepatic mass, as found in our patient, has not been described in the literature thus far. 

In previous studies, ectopic adrenal tissue has been found in the following areas: celiac axis region (32%), broad ligament (23%), testis (7.5%), and spermatic cord (1-9.3%) [3]. Because they both originate from the mesoderm, the kidneys (0.1-6%) and liver [4] may be sites of adrenal ectopy. Ectopic adrenal tissue has been found in the placenta, lungs, and cerebral cavity in some cases [5,6]. Case reports on functional characteristics have shown that hypersecretion can occur in all adrenal cortex layers. Cushing’s syndrome [12] or primary aldosteronism [13] has been reported in cases of ectopic adrenal tissue, similar to findings for normal adrenal glands. However, only one case of pheochromocytoma arising from ectopic adrenals has been reported in Japan [9]. Here, we report a case of an immunohistochemically confirmed pheochromocytoma arising from ectopic adrenal tissue. To the best of our knowledge, only one similar case has been described in the literature [9]. 

The therapeutic management of ectopic and ectopic pheochromocytomas is similar. Indeed, our patient underwent an adrenalectomy. Some reports recommend surgeons to resect lesions suspicious of ectopic adrenal tissue during surgery [14,15], whereas others suggest that it is important to diagnose ectopic adrenal tissue as non-invasively as possible considering that they require no treatment due to their asymptomatic nature [16].

Patients diagnosed with pheochromocytomas and paragangliomas should undergo comprehensive genetic testing as part of their standard care. It is crucial to identify specific genetic mutations that may be linked to the development of multifocal recurrent (SDHx, HIF2A/PHD1/PHD2, and FH mutations) or metastatic pheochromocytomas and paragangliomas (SDHB, MAX, and FH mutations) as well as other non-chromaffin cell tumors (HIF2A mutations) [17-19]. Therefore, comprehensive genetic testing in a sequential manner is important.

## Conclusions

Ectopic adrenal tissue is usually asymptomatic and contains only the adrenal cortex. However, the adrenal medulla can be found when the ectopic adrenal tissue is in close proximity to the adrenal glands. Adrenal tumors can form from ectopic adrenal tissue in the same way as they do in eutopic adrenal glands. 

The formation of pheochromocytomas from ectopic adrenal tissue is uncommon, and they are treated similarly to eutopic tumors. Only one case report has shown a pheochromocytoma arising from ectopic adrenal medulla tissue in Japan.
